# P-2098. Clinical culture rates among hospitalized patients in the United States, 2012-2021

**DOI:** 10.1093/ofid/ofae631.2254

**Published:** 2025-01-29

**Authors:** Hannah Wolford, Natalie McCarthy, James Baggs, Sujan Reddy, Joseph D Lutgring

**Affiliations:** CDC, Atlanta, Georgia; CDC, Atlanta, Georgia; CDC, Atlanta, Georgia; CDC, Atlanta, Georgia; Division of Healthcare Quality Promotion, Centers for Disease Control and Prevention, Atlanta, GA

## Abstract

**Background:**

Microbial cultures are used in clinical care and to track trends in infectious diseases. Changes in culturing practices may affect these trends but have not been thoroughly explored. This analysis examines temporal and facility variability of clinical cultures among a cohort of acute care hospitals in the U.S. from 2012-2021.

**Figure:**
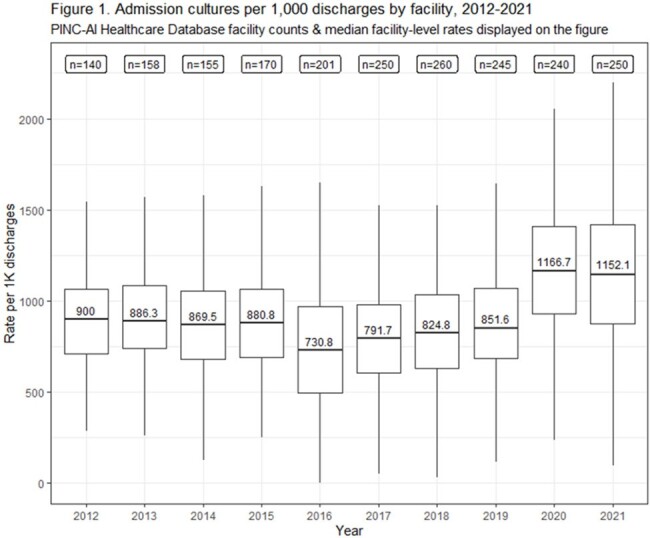

**Methods:**

We used microbiology data from a dynamic cohort of hospitals reporting data to the PINC-AI Healthcare Database from 2012-2021. Surveillance cultures and hospitals with outlier hospital-months were excluded. We defined admission cultures as those collected on or before calendar day 3 of hospitalization and post-admission cultures as those collected after calendar day 3. Admission culture rates were calculated per 1,000 discharges. Post-admission culture rates were calculated per 1,000 patient days. We calculated overall rates as well as rates stratified by specimen source (blood, respiratory, urine, other sterile sites, and other non-sterile sites). To account for multiple blood culture sets, only the first blood culture per patient per day was counted in this analysis.

**Figure:**
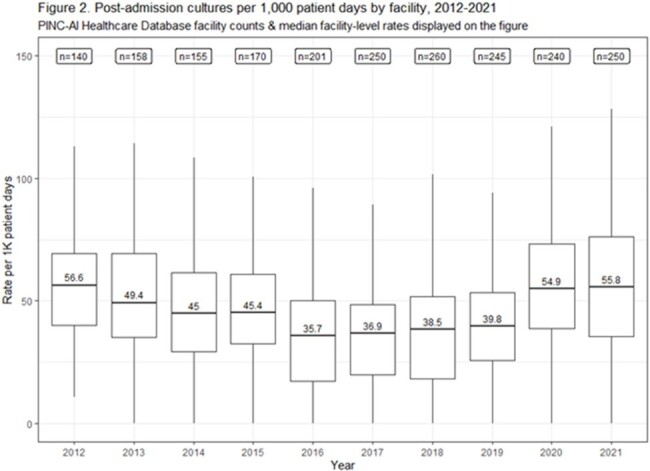

**Results:**

From 2012-2021, 361 hospitals were included in our cohort. Admission culture rates remained stable from 2012 through 2019. Admission culturing rates and facility variability increased in 2020 and 2021 (Fig. 1). Post-admission culture rates decreased from 2012 to 2016 and then plateaued; increases in rates and facility variability were noted in 2020 and 2021 (Fig. 2). Admission blood cultures increased over time and admission urine cultures decreased; respiratory and other non-sterile site cultures increased in 2020 and 2021 (Fig. 3). Most types of post-admission cultures declined from 2012-2016; post-admission blood cultures increased between 2016-2021 (Fig. 4).

**Figure:**
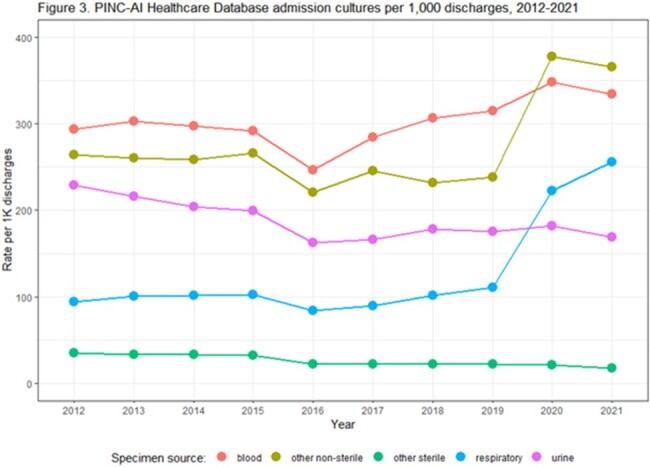

**Conclusion:**

Both admission and post-admission cultures increased during the early years of the COVID-19 pandemic, and these increases were driven by respiratory, other non-sterile, and blood cultures (which increased since 2016). Prior to 2020, culturing trends differed by specimen type and timing of culture. Assessing factors that contribute to testing tends and facility variability can inform diagnostic stewardship efforts and increase understanding of how testing changes may impact infection rates.

**Figure:**
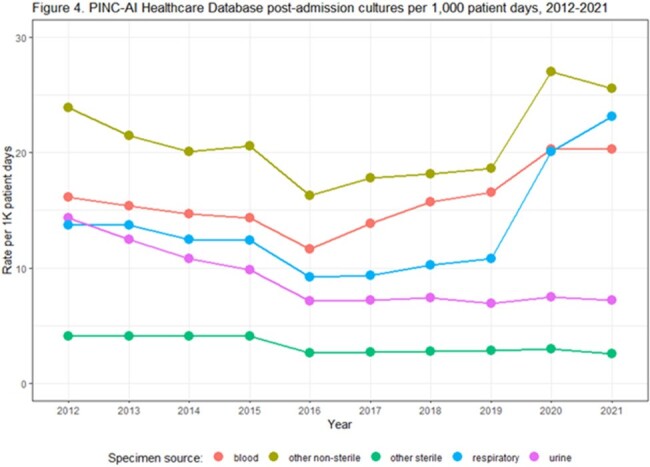

**Disclosures:**

All Authors: No reported disclosures

